# New Anæsthetics

**Published:** 1849-06

**Authors:** 


*New Anæsthetics.* By Thomas Nunneley.—Though my paper "On Anæsthesia, and the Agents by which it may be produced," will appear in the forthcoming part of the next volume of the *Transactions of the Provincial Medical and Surgical Association,* now in the press, yet as some little time will necessarily elapse before it can be published, it may not be improper, nor without interest, to state, that amongst the many substances upon which I have experimented, there are two which are most worthy of attention, as of easy practical application.

The one, which was amongst the earliest I tried, *is common coal gas.* It is a safe, manageable, and effective anæsthetic, and very cheap, as every body knows; though the smell is at first unpleasant, it is inhaled without difficulty or repugnance.

The second is a substance which I have more recently discovered, and if my anticipations be well founded, it will be found to be the best agent yet mentioned, and will, I think, supersede those now employed.

I believe it to be possessed of all the good properties of chloroform, and in a great degree free from those which are objectionable. It is equally pleasant, potent, and speedy in its action. The anæsthesia produced by it may be rendered as profound and prolonged as may be wished. While a smaller quantity of it than of chloroform, will produce a sufficient degree of insensibility, a larger quantity may be given with impunity. The state of collapse is not so great. The animal may be recovered from a more deadlike condition, than where this is induced by chloroform; at the same time, the process of recovery is more rapid, and it is unattended by any of those distressing symptoms so often witnessed in animals rallying from a large dose of chloroform.

The substance is the *chloride of olefiant gas,* as named in "Fowne's Manual;" the hydrochlorate of *chloride of acetyle, or oil of olefiant gas,* in the eighth edition of "Turner's Chemistry;" and formerly called *Dutch oil,* or *oil of the Dutch chemists.*

In appearance and smell it is not very dissimilar from chloroform, but in composition it differs most materially. Chloroform being composed of two atoms of carbon, one of hydrogen, and three of chlorine, with a boiling point of 140°, the specific gravity of the liquid being 1·480°, of the vapor, 4·2°; while the chloride of olefiant gas is composed of four atoms of carbon, four of hydrogen, and one of chlorine; its boiling point is 180°, the specific gravity of the liquid 1·247°; of the vapor 3·5484°; constituting differences which are very important, and sufficient, I believe, to explain the facts of its superiority.

I need not here detail any of the experiments upon which this statement is founded, nor the reasons which induced me to make trial of this and many other substances, as they are mentioned at length in the paper to which I have alluded, and which will shortly be in the hands of the members of the Association, my object now being simply to mention the fact. —*Provincial Medical & Surgical Journal.*

